# Position Estimator Design for a MEMS Top-Drive Electrostatic Rotary Actuator

**DOI:** 10.3390/s20247081

**Published:** 2020-12-10

**Authors:** Jemin Woo, Bongsu Hahn, Changsun Ahn

**Affiliations:** 1 School of mechanical engineering, Pusan National University, Busan 46241, Korea; woojmn@pusan.ac.kr; 2 Department of robotics engineering, Kyungil University, Gyeongsan 38428, Korea; hahn@kiu.kr

**Keywords:** electrostatic rotary actuator, MEMS, position estimation, unscented Kalman filter

## Abstract

The capacitance and rotor angle of a MEMS top-drive electrostatic rotary actuator do not have a linear relationship due to the non-ignorable fringe effect and low aspect ratio of the electrodes. Therefore, the position estimation is not as straightforward as that for a comb-drive linear actuator or a side-drive rotary actuator. The reason is that the capacitance is a nonlinear and periodic function of the rotor angle and is affected by the three-phase input voltages. Therefore, it cannot be approximated as a simple two-plate capacitor. Sensing the capacitance between a rotor and a stator is another challenge. The capacitance can be measured in the electrodes (stators), but the electrodes also have to perform actuation, so a method is needed to combine actuation and sensing. In this study, a nonlinear capacitance model was derived as a data-driven model that effectively represents the nonlinear capacitance with sufficient accuracy. To measure the capacitance accurately, the stator parts for actuation and those for sensing are separated. Using the nonlinear model and the capacitance measurement, an unscented Kalman filter was designed to mitigate the large estimation error due to the periodic nonlinearity. The proposed method shows stable and accurate estimation that cannot be achieved with a simple two-plate capacitor model. The proposed approach can be applied to a similar system with highly nonlinear capacitance.

## 1. Introduction

Since the introduction of microelectromechanical systems (MEMS), research on MEMS rotary actuators has received great interest [1,2,3]. MEMS rotary actuators are essential parts of equipment for various applications, such as micro-automation equipment, biomedical equipment, ultra-precision measuring equipment, and optical equipment. Possible applications include the calibration of small gyro sensors, azimuth control using an optical mirror, scanning angle adjustment in a display device, and camera focusing at the end of an endoscope [3,4,5].

Electrostatic MEMS actuators are widely used as rotary actuators because of their simple design and low power consumption [3,6,7]. The electrostatic actuator operates using the electrostatic force generated by electric charges stored between a rotor and a stator. There are various types of MEMS electrostatic rotary actuators. Depending on the type of drive, they can be divided into top-drive [2], side-drive [8], and wobble drive actuators [9]. The comb actuator [10,11,12] is the best-known electrostatic actuator and mainly uses the side-drive method.

The side-drive method has an advantage of high rotor stability, but its torque is limited due to the small lateral area. Top-drive actuator has a comparably lower stability due to the vertical force between the rotor and stator, but the large area of electrodes results in large torque. Therefore, if the stability and transmission of stable torque in the actuator are guaranteed, it would have many advantages over other drive types. 

For the stability of the rotor and torque transmission, studies have applied solid [13,14], liquid [15,16], and gas bearings [17,18] between the rotor and the stator. The conductor-ring liquid bearing allows electrical connection between the rotor and stator to make up for shortcomings of the solid bearing and gas bearing. The actuator considered in this study is a top-drive electrostatic rotary actuator equipped with a ring-shaped bearing.

Popular research topics in MEMS rotary actuator studies include the selection of drive type, methods for stable transmission of rotational force, position sensing, and control for precision positioning. For precision positioning, accurate sensing is essential, particularly when there are disturbances and model uncertainties [7,11,19]. The most general sensor for a rotary actuator is an encoder, but installing an encoder in a MEMS actuator is almost impossible due to the space limitation. Therefore, position and velocity information are not directly measured but estimated using the measured capacitance [20,21]. For state estimation, an observer is usually used. Accurate system modeling, proper selection of measurable signals, and the design of corresponding measurement systems are crucial for accurate state estimation using an observer.

For control and estimation, MEMS electrostatic actuators are usually modeled as a flat plate capacitor [11,12,22]. However, a simple two-plate capacitor model is no longer valid for MEMS electrostatic actuators that have a wide movement range and a small aspect ratio of electrodes, such as MEMS rotary actuators. The reason is that the fringe effect cannot be ignored due to the small aspect ratio [23,24], and more than two electrodes can be close to each other during the rotational motion. These characteristics result in a highly nonlinear electric field, so a simple two-plate capacitor model is invalid, which makes estimating and controlling the position of the MEMS rotary actuator difficult. There are simple analytical models that can effectively represent the fringe effect, but this approach cannot handle multiple electrodes that are close to each other [2,23,25].

Capacitances are usually measured to estimate the rotor position in most MEMS electrostatic actuators. Capacitance sensing is based on the frequency responses of an electric circuit with a capacitor. To sense the capacitance, sinusoidal voltage excitation is applied to some electrodes, and the voltage differences across resistances are measured. A voltage input to generate torque is also applied to the electrodes, so two different kinds of voltage inputs can be applied to the same electrodes.

There are multiple ways to apply the two voltage inputs to the electrodes. One is applying them to the same electrode at the same time [7,20], which is achieved by separating the low-frequency driving signals and the high-frequency sensing signal. This method is cost-effective and convenient for MEMS devices because of the simple sensing configuration. However, it is difficult to obtain a clear sensing signal through frequency separation due to the difficulty of complete bandwidth separation. Another way is applying the two voltage inputs to the same electrode but during different times using an electrical switch [26]. This has an advantage in that the measured sensing signal is independent of the driving signal, but there is a problem in that fast electrical switching causes non-ideal characteristics.

This study deals with a top-drive MEMS electrostatic rotary actuator that has three-phase input voltage. It has an infinite moving range and a small aspect ratio of the electrodes, so the fringe effect and multiple electrodes should be considered. Furthermore, three electrodes can be used to excite the capacitance systems to measure the effect of capacitance variations, which is different from a system that can be approximated using a simple two-plate capacitor model. 

In this research, we propose a measurement system and accurate observer design for position estimation. For accurate estimation, the driving electrodes and sensing electrodes are separated, and an RC circuit is used to measure the effect of capacitance variations corresponding to the rotor position variations. As the observer model, data-driven models are used to effectively reflect the highly nonlinear characteristics caused by the fringe effect and multiple electrodes. The best selection of an excitation method and the corresponding measurement model are proposed to measure the effect of capacitance variations. An unscented Kalman filter was used to mitigate a large estimation error that occurs when the nonlinearity of capacitor increases. The proposed estimator and sensing methodology were validated by a simulation performed using COMSOL and MATLAB/Simulink. 

## 2. MEMS Electrostatic Rotary Actuator 

**Figure 1:**
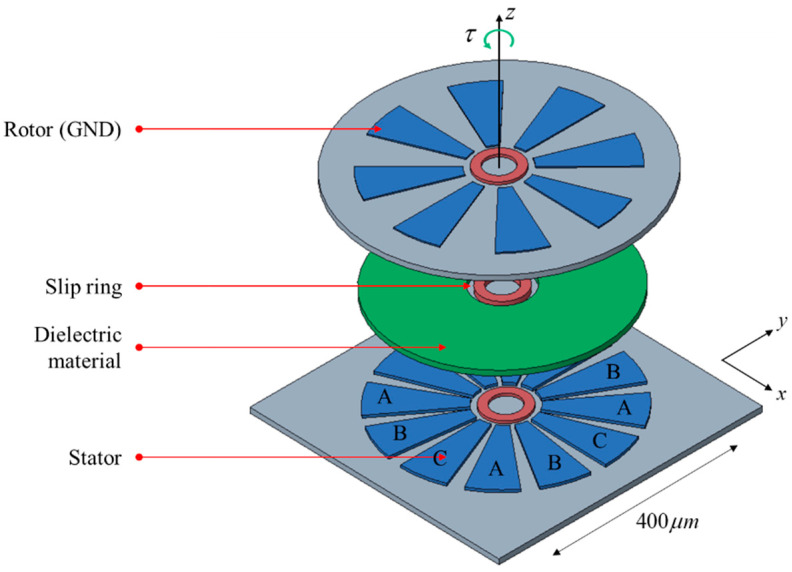
Top-drive electrostatic rotary actuator.

Figure 1 shows a diagram of the electrostatic rotary actuator, which consists of electrodes for the rotor and stator, dielectric material, and a slip ring. The slip ring is a bearing on which the rotor rotates freely about a fixed axis and stably transmits electrostatic torque. The dielectric material increases the capacitance and prevents the movement of electrons between the stator and the rotor. Another role of the dielectric material is preventing the rotor and stator from sticking by maintaining a gap between them. 

The rotor and stator are conducting electrodes. The rotor is electrically grounded, and input voltage is applied through the stator. The stator consists of 12 poles with three phases (A, B, and C.) The actuator can be driven using three-phase 12-pole electrostatic conversion.

**Figure 2:**
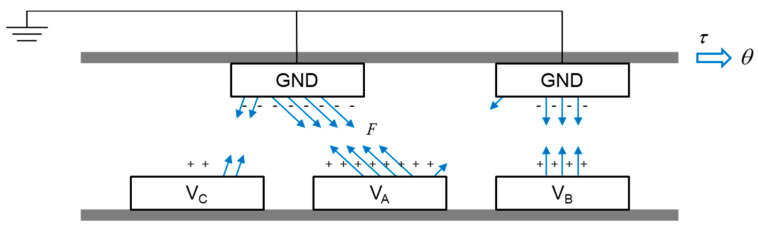
Principle of the electrostatic rotary actuator operations.

Figure 2 presents the actuation principle of the electrostatic rotary actuator. When three-phase input voltages are supplied to the poles of the stator, positive electric charges are accumulated on the stator, and negative electric charges are accumulated on the rotor. As a result, electrostatic force is generated between the electrodes, so the rotor moves. As the rotor rotates, the overlapped area between the rotor and stator changes, so the amount of electric charge and corresponding capacitance change. 

**Figure 3:**
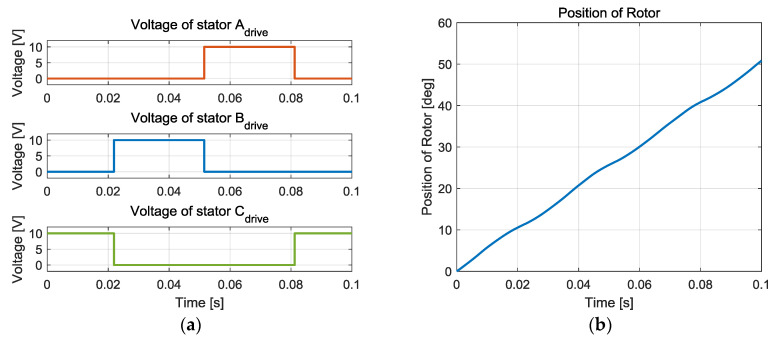
Example of open loop operation: (**a**) Input voltage, (**b**) Rotor position.

**Figure 4:**
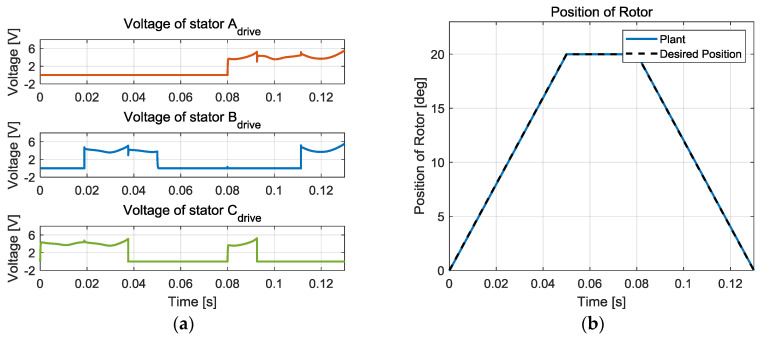
Example of position control operation: (**a**) Input voltage, (**b**) Rotor position.

Figure 3 and Figure 4 show examples of actuator operations. The input voltage can be a simple PWM signal. To rotate the rotor in one direction without changing the sign of the angular velocity, the voltage should be supplied to one or two phases according to the angle because the sign of the electrostatic torque is determined by rotor position. Open-loop input with constant voltage results in increasing the angle, as shown in Figure 3, where the rotor velocity is not constant even for constant voltage input due to nonlinearity of the force generation of the capacitor.

The angular position can be controlled by adjusting the magnitude of the voltage, so feedback is necessary. Figure 4 shows an example of position control where the rotor follows a desired position. All three phases are involved for the position tracking operations. In this example, it is assumed that a PID controller with perfect angle measurement is used. For position control, angle information is essential. The capacitance changes as the position of the rotor relative to the stator changes, so the capacitance can be used in estimating the rotor position.

## 3. Scheme for Simultaneous Operation and Capacitance Sensing of Electrostatic Actuator

### 3.1. Input Voltage Supply Strategy

The MEMS-based electrostatic actuators do not have space for a rotary encoder. Therefore, the rotor position is estimated based on the measured capacitance, which is a function of the rotor position. The capacitance sensing is based on the frequency responses of the RC system. To sense the capacitance, a sinusoidal voltage excitation is applied to some electrodes, and the voltage differences across resistances (sensing voltage) are measured. Therefore, we can apply two different kinds of voltage inputs: a voltage input to generate torque (driving voltage) and a voltage to excite the RC circuit to sense the capacitance (sensor excitation voltage). There are a few ways to apply the two voltage inputs to the electrodes: applying the two voltage inputs to the same electrode at the same time and applying the two voltage inputs to the same electrode at different times. 

The first method has advantages in that all electrodes can be involved, so it maximizes the torque. However, filtering is required in the sensing system because the sensing voltage contains the mixed responses of the driving voltage and sensor excitation voltage, which may reduce the estimation accuracy, particularly when the driving signal has a high frequency component due to feedback control. The second method does not have the issues of mixed signals, but control and sensing cannot be performed simultaneously, and the maximum torque is lower. 

**Figure 5:**
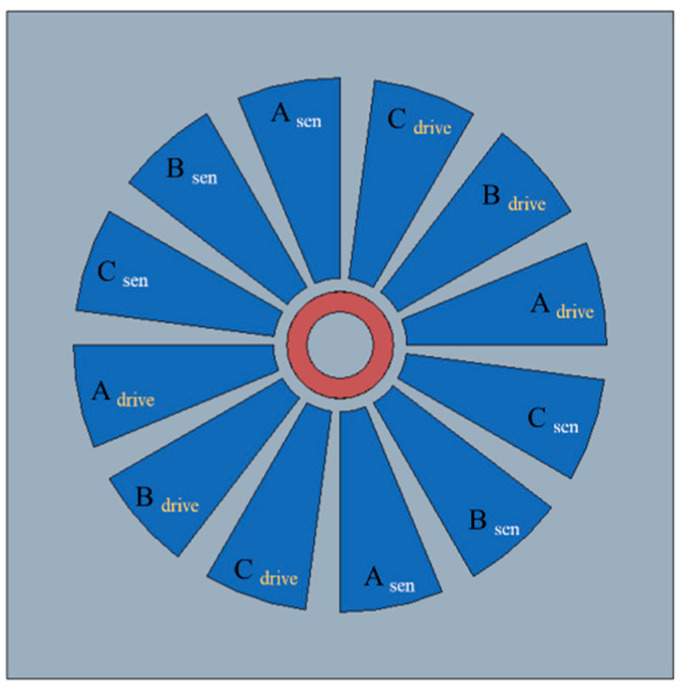
Input voltage supply strategy for simultaneous driving and sensing.

In the proposed method, the two voltage inputs are applied to separate electrodes at the same time, as shown in Figure 5. This method does not have the issues of mixed signals, and control and sensing can be performed simultaneously, but the maximum torque is lower. However, the reduced maximum torque is not critical because the torque limit of a top-drive rotary actuator is much higher than that of a side-drive rotary actuator. 

For this voltage supply strategy, each phase of the stator is divided into two parts: one for driving and one for sensing. They consist of A_drive_, B_drive_, C_drive_, A_sen_, B_sen_, and C_sen_, all of which are in the actuator and can be driven without tilting by forming a symmetrical structure. is much smaller than the driving voltages (tens of volts), so the sensing voltage inputs do not affect the operation.

### 3.2. RC Circuit for Sensing the Effect of Position Variation

**Figure 6:**
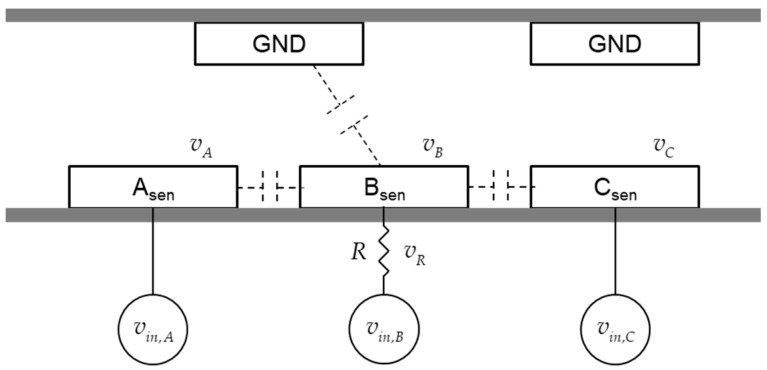
RC circuit construction for capacitance measurement with the voltage of three phases.

To measure the capacitance, the RC circuit is configured by connecting a resistor to the stator [7]. In Figure 2, the amount of electric charge in an electrode depends on not only the voltage applied to the electrode but also the voltages of neighboring electrodes. Figure 6 presents a 2-D view of the cylindrical structure of the actuator. The capacitor-sensing system consists of three electrodes where excitation voltage inputs are applied as well as a resistor to measure the response voltage of the RC circuit. A resistor is attached to one of the three-phase stators, and the other phase is directly connected to the power supply.

There are three places where the voltage inputs can cause variation of the capacitance or accumulated electric charge in electrode *B_sen_*, as shown in Figure 6. The three excitation voltage inputs are sinusoidal as follows:

(1) $$\begin{array}{l} v_{in,A}(t)=V_{in,A}\sin(\omega_{1}t),\;\;\\ v_{in,B}(t)=V_{in,B}\sin(\omega_{2}t),\;\\ v_{in,C}(t)=V_{in,C}\sin(\omega_{3}t). \end{array}$$

When the magnitude of the excitation voltage is sufficiently small, the corresponding output voltage measured in the resistance *R* is:

(2) $$v_{R}(t)=V_{R1}\sin(\omega_{1}t+\phi_{1})+V_{R2}\sin(\omega_{2}t+\phi_{2})+V_{R3}\sin(\omega_{3}t+\phi_{3}).$$

The magnitudes of the voltage output are mainly affected by the differential capacitance, which is determined by the rotor position. Therefore, the rotor position can be estimated by measuring *v_R_*.

## 4. System Model for Estimator Design

### 4.1. Rotor Dynamics

**Figure 7:**
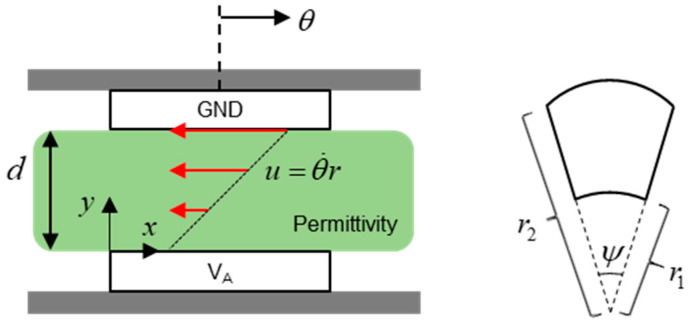
The viscous resistance caused by dielectric material.

When input voltage is supplied to the stators, torque is generated, and the rotor rotates. The frictional resistance is generated by the dielectric material as the rotor rotates. Figure 7 explains the viscous friction resistance in the dielectric material.

The viscous friction torque is caused by shear force and is a function of the coefficient of viscosity of the dielectric material, the area of the rotor, the radius of the rotor, and the vertical derivative of the angular velocity:

(3) $$\begin{array}{l} \tau_{friction}=\int_{0}^{\psi }\int_{r_{1}}^{r_{2}}\sigma r\cdot rdrd\phi =\int_{0}^{\psi }\int_{r_{1}}^{r_{2}}\mu \frac{\dot{\theta }r}{d}r\cdot rdrd\phi =\frac{\psi \mu \dot{\theta }}{d}\int_{r_{1}}^{r_{2}}r^{3}dr\\ \;\;\;\;\;\;\;\;\;\;\;\;\;=\frac{\psi \mu \dot{\theta }\left(r_{2}{}^{4}-r_{1}{}^{4}\right)}{4d}=\frac{\psi \left(r_{2}{}^{2}-r_{1}{}^{2}\right)}{2}\frac{\mu \dot{\theta }}{d}\frac{\left(r_{2}{}^{2}+r_{1}{}^{2}\right)}{2}\\ \;\;\;\;\;\;\;\;\;\;\;\;=A\frac{\mu \dot{\theta }}{d}\frac{\left(r_{2}{}^{2}+r_{1}{}^{2}\right)}{2} \end{array}$$

where *I_z_* is the inertia of the rotor, *τ_friction_* is the friction torque, *μ* is the coefficient of viscosity, *A* is the area of the rotor, $\dot{\theta }$ is the angular velocity of the rotor, *d* is the distance between the stator and rotor, *r*_1_ is the inner radius of the rotor, and *r*_2_ is the outer radius of the rotor. The rotational dynamics of the rotor are expressed as:

(4) $$\tau -\tau_{friction}=I_{z}\cdot \ddot{\theta }$$

where *I_z_* is the inertia of the rotor, and *τ* is the driving torque.

### 4.2. Torque Model

When a voltage is applied to the electrodes, the electrodes are charged. The amount of charge of each electrode is dependent on the voltage of the electrode, the voltage levels of the neighbor electrodes, and the rotor position. Therefore, the generated torque is expressed as follows:

(5) $$\tau (V_{A},V_{B},V_{C},\theta )$$

The most common way of modeling the force or torque generation is by deriving an equation based on a simple flat capacitor model. However, as mentioned, this top-drive electrostatic rotary actuator cannot be approximated by a simple flat capacitor. For a capacitor in which the electrode shape is not a plate, some methods have been proposed to derive an analytical model. For example, the electric field of a complex-shape capacitor has been analyzed using Schwarz–Christoffel mapping (SCM) [27,28,29,30]. However, these methods can only be applied when the capacitor consists of only two electrodes. There is no simple analytical model for a capacitor consisting of more than two electrodes. 

**Figure 8:**
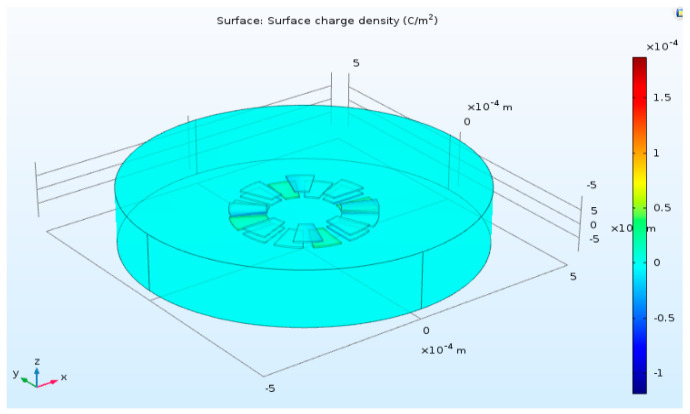
Rotary actuator model for torque and electric charge analysis.

In this research, we propose a data-driven model for the torque function. COMSOL multi-physics software was used to calculate the torque. The simulation model is shown in Figure 8. The electrodes that include the stators and rotors are in a sector form with the angle of 22.5 degrees. The interval angle between each stator is 7.5 degrees and the interval angle between each rotor is 22.5 degrees. The inner radius of each electrode is 100 μm and the outer radius is 200 μm. The distance between stators and rotors is 15 μm. For COMSOL simulation setup, the radius of the boundary space is 500 μm and height is 200 μm. The material of electrodes is copper and their density is 8700 kg/m^3^ and electrical conductivity is 5.998 × 10^7^ S/m. The dielectric material is poly methyl methacrylate (PMMA) with liquid state, and its relative permittivity is 3.0 and density is 1190 kg/m^3^. The material of the boundary space is air, its relative permittivity is 1.0 and electrical conductivity is zero.

**Figure 9:**
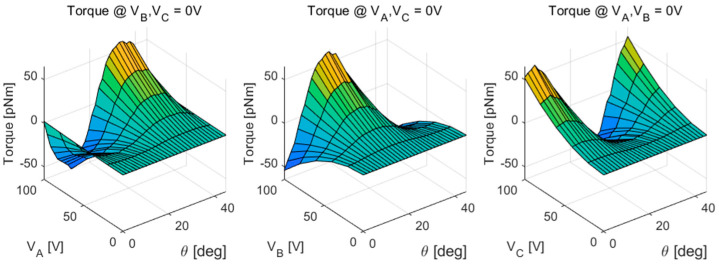
The torque model.

The sequence type of mesh is physics-controlled mesh and the element size is extra fine. By simulating electrostatic rotary actuator in all conditions of the rotor position and voltage inputs, the torque model was derived. The range of the rotor position for one period is from 0 to 45 degrees, the electrodes of the rotor are grounded (the electric potential is 0 V), and the voltage range supplied to the electrodes of the stator is 0 to 100 V. Figure 9 shows the derived torque model. The torque model is periodic form for the rotor position because of the repetitive structure of the stators and rotors. 

### 4.3. Capacitance Model

**Figure 10:**
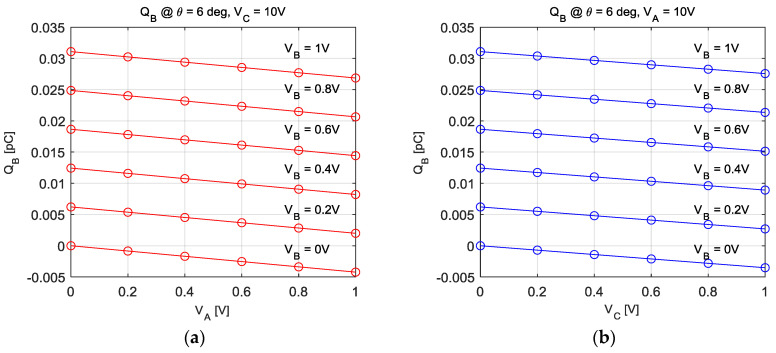
The amount of electric charges on the rotor when supplying (**a**) V_A_ and V_C_, (**b**) V_B_ and V_C_.

**Figure 11:**
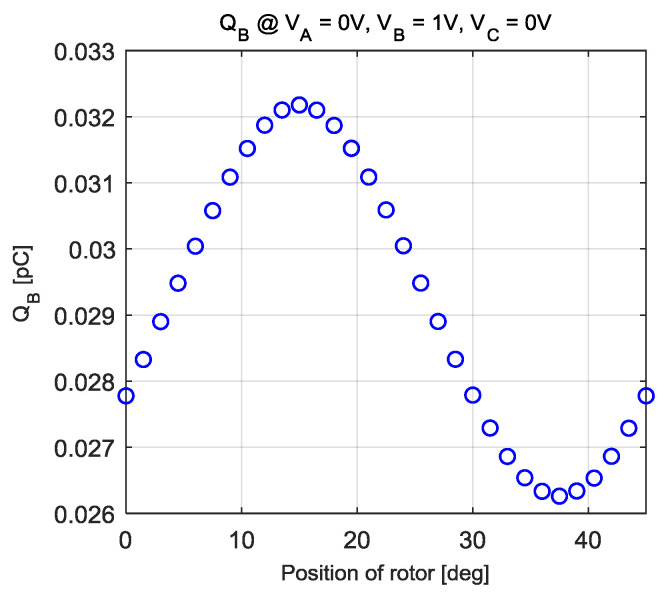
The amount of electric charges on the rotor when the three-phase voltage is supplied with a fixed voltage.

The capacitance model was derived in a similar way to the torque model derivation. Figure 10 and Figure 11 show some of the results from the COMSOL analysis. In both graphs in Figure 10, the electric charges are linear with respect to the voltage inputs. The electric charges can be expressed as a linear combination of the three voltage inputs *V_A_*, *V_B_*, and *V_C_*. Figure 11 presents the amount of electric charge as the rotor position increases, which is a periodic nonlinear function of the rotor position. 

The electric field generated by electrodes located far from the electrode of interest is too weak to consider, and the electric charges in the neighbor electrodes dominate the electric field. Therefore, the amount of electric charge in the electrode *B_sen_* of the stator shown in Figure 6 is expressed as follows:

(6) $$Q_{B}(V_{A},V_{B},V_{C},\theta )=f_{A}(\theta )\cdot V_{A}+f_{B}(\theta )\cdot V_{B}+f_{C}(\theta )\cdot V_{C}.$$

The gradient functions *f_A_*(*θ*), *f_B_*(*θ*), and *f_C_*(*θ*) are partial derivatives of the electric charges with respect to voltage:

(7) $$f_{A}(\theta )=\frac{\partial Q_{B}}{\partial V_{A}},\;\;\;f_{B}(\theta )=\frac{\partial Q_{B}}{\partial V_{B}},\;\;\;f_{C}(\theta )=\frac{\partial Q_{B}}{\partial V_{C}}.$$

Therefore, *f_A_*(*θ*), *f_B_*(*θ*), and *f_C_*(*θ*) mean the differential capacitances. The model of the differential capacitance can be numerically derived from the function of *Q_B_*. The function of the differential capacitance has a periodic form, so it is approximated using a Fourier series as follows.

(8) $$\begin{array}{l} f_{A}(\theta )=c_{0}+\sum_{k=1}^{5}\left[d_{i}\cos(k\cdot 8\theta )+e_{i}\cos(k\cdot 8\theta )\right],\\ f_{B}(\theta )=a\cos(8\theta )+b,\\ f_{C}(\theta )=f_{A}(\theta +\frac{\pi }{12}). \end{array}$$

where *a* = 2.9405 × 10^−15^, *b* = 3.0051 × 10^−14^, *c_0_* = −3.9430 × 10^−14^, *d*_1_ = −2.0942× 10^−16^, *d*_2_ = 5.9075 × 10^−17^, *d*_3_ = −5.5794 × 10^−18^, *d*_4_ = −3.0530 × 10^−18^, *d*_5_ = 1.8782 × 10^−18^, *e*_1_ = −3.5425 × 10^−16^, *e*_2_ = −1.0049 × 10^−16^, *e*_3_ = 4.6715 × 10^−19^, *e*_4_ = −5.0257 × 10^−18^, *e*_5_ = −1.4881 × 10^−18^.

**Figure 12:**
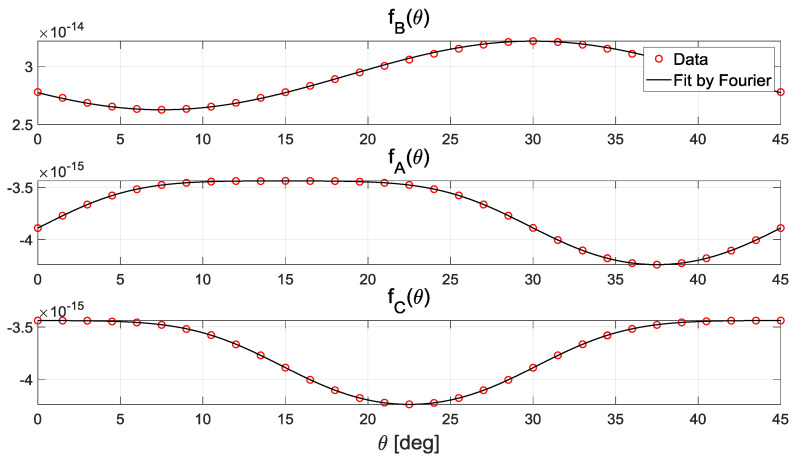
The amount of electric charges on the rotor when the three-phase voltage is supplied with a fixed voltage.

The numerically derived function of the differential capacitances and their fitting functions are shown in Figure 12. The differential capacitances are functions of the rotor position. Therefore, we can estimate the position once the differential capacitance is measured. The magnitude of the differential capacitance corresponding to *V_B_* is 10 times larger than the others. Therefore, the measured capacitance excited by *V_B_* is more robust than the others because the signal-to-noise ratio of *f_B_*(*θ*) is larger than those of *f_A_*(*θ*) and *f_C_*(*θ*). Furthermore, *f_A_*(*θ*) and *f_C_*(*θ*) show ranges of zero sensitivity where the position cannot be estimated. Therefore, measuring *f_B_*(*θ*) for measuring *f_B_*(*θ*)-related signals would be beneficial for achieving robust and accurate estimation.

### 4.4. Measurement Signal and Measurement Model

The measurement system shown in Figure 6 can be modeled as an electric circuit with a nonlinear capacitor as follows:

(9) $$\begin{array}{l} v_{A}=v_{in,A},\;\;\;v_{B}=v_{in,B}-v_{R},\;\;\;v_{C}=v_{in,C},\;\;\;v_{R}=Ri,\;\;\;\;\;\frac{dQ_{B}}{dt}=i,\;\;\\ Q_{B}=Q_{B}(v_{A},\;\;v_{B},\;\;v_{C},\;\theta ). \end{array}$$

For small perturbation around the equilibrium point, the equation can be linearized as follows:

(10) $$\begin{array}{l} \delta v_{A}=\delta v_{in,A},\;\;\delta v_{B}=\delta v_{in,B}-\delta v_{R},\;\;\;\;\delta v_{C}=\delta v_{in,C},\;\;\delta v_{R}=R\delta i,\;\;\;\frac{d\delta Q_{B}}{dt}=\delta i,\;\\ \delta Q_{B}=\frac{\partial Q_{B}}{\partial v_{A}}\delta v_{A}+\frac{\partial Q_{B}}{\partial v_{B}}\delta v_{B}+\frac{\partial Q_{B}}{\partial v_{C}}\delta v_{C}+\frac{\partial Q_{B}}{\partial \theta }\delta \theta \\ \;\;\;\;\;\;\;\;\;\;=f_{A}(\theta )\delta v_{A}+f_{B}(\theta )\delta v_{B}+f_{C}(\theta )\delta v_{C}+\frac{\partial Q_{B}}{\partial \theta }\delta \theta . \end{array}$$

The resulting dynamic equation is:

(11) $$\frac{1}{R}\int \delta v_{R}\;\;dt+f_{B}\delta v_{R}=f_{A}\delta v_{in,A}+f_{B}\delta v_{in,B}+f_{C}\delta v_{in,C}+\frac{\partial Q_{B}}{\partial \theta }\delta \theta ,$$

where the output signal is *δv_R_*, and the input signals are *δv_in,A_*, *δv_in,B_*, *δv_in,C_*, and *δθ*. The transfer functions from the controllable input signal to the output signal are:

(12) $$\frac{\Delta V_{R}(s)}{\Delta V_{in,A}(s)}=\frac{f_{A}Rs}{1+f_{B}Rs},\;\;\;\;\;\;\frac{\Delta V_{R}(s)}{\Delta V_{in,B}(s)}=\frac{f_{B}Rs}{1+f_{B}Rs},\;\;\;\;\;\;\frac{\Delta V_{R}(s)}{\Delta V_{in,C}(s)}=\frac{f_{C}Rs}{1+f_{B}Rs}.$$

From the transfer functions, we can derive the signal amplification ratio as follows:

(13) $$\begin{array}{l} \frac{\left|\Delta V_{R}(j\omega )\right|}{\left|\Delta V_{in,A}(j\omega )\right|}=\frac{f_{A}(\theta )R\omega }{\sqrt{1+f_{B}{\left(\theta \right)}^{2}R^{2}\omega^{2}}},\;\;\;\;\;\\ \frac{\left|\Delta V_{R}(j\omega )\right|}{\left|\Delta V_{in,B}(j\omega )\right|}=\frac{f_{B}(\theta )R\omega }{\sqrt{1+f_{B}{\left(\theta \right)}^{2}R^{2}\omega^{2}}},\;\;\;\;\;\\ \frac{\left|\Delta V_{R}(j\omega )\right|}{\left|\Delta V_{in,C}(j\omega )\right|}=\frac{f_{C}(\theta )R\omega }{\sqrt{1+f_{B}{\left(\theta \right)}^{2}R^{2}\omega^{2}}}. \end{array}$$

The left terms of (13) can be computed from the excitation signals and measured signals. The right terms of (13) can be computed using the differential capacitor model (8) if *θ* is known. If the left terms are known but *θ* is unknown, then *θ* can be determined using one of the equations of (13). Therefore, the right terms of (13) can be the measurement model for the observer design, and we will treat the signal amplification ratio as the measurement signal.

## 5. Estimator Design

The rotary actuator system can be expressed in the following discrete form:

(14) $$\begin{array}{l} x_{k}=F_{k-1}x_{k-1}+G_{k-1}u_{k-1}+w_{k-1}\\ z_{k}=h(x_{k})+v_{k}\\ F=\left[\begin{array}{cc} 1 & \Delta t\\ 0 & 1-\frac{\mu A(r_{1}{}^{2}+r_{2}{}^{2})}{2dI_{z}}\Delta t \end{array}\right],\;\;G=\left[\begin{array}{c} 0\\ \frac{1}{I_{z}}\Delta t \end{array}\right],\;\;\;\;x=\left[\begin{array}{c} \theta \\ \dot{\theta } \end{array}\right],\;\;\;u=\tau ,\;\;\\ z=\frac{\left|\Delta V_{R}(j\omega )\right|}{\left|\Delta V_{i}(j\omega )\right|},\;\;h(x)=\frac{f_{i}(\theta )R\omega }{\sqrt{1+f_{B}{\left(\theta \right)}^{2}R^{2}\omega^{2}}},\;\;\;\;i=A,B,C. \end{array}$$

where *w* is the process noise, and *v* is the observation noise. The model of the system dynamics is linear, but the measurement model is nonlinear. To deal with the nonlinear measurement model, we considered an extended Kalman filter (EKF) [31] and unscented Kalman filter (UKF) [32,33].

In EKF design, there are two main design factors: *Q*, the covariance matrix of the process noise; and *R*, the covariance matrix of the measurement noise. *Q* and *R* for the EKF for the rotor position estimator were set as follows:

(15) $$Q=\left[\begin{array}{cc} 0.001 & 0\\ 0 & 0.091 \end{array}\right],\;\;\;R_{A}=1.6\times 10^{-6},R_{B}=5.97\times 10^{-5},R_{C}=1.6\times 10^{-6}.$$

The UKF does not need linearization and propagate mean and covariance information through nonlinear transformations. The UKF operates with sample points generated based on a Gaussian random variable instead of using linearization. The design factors of the UKF are *Q*, *R*, and *W_i_* and were set as follows:

(16) $$\begin{array}{l} Q=\left[\begin{array}{cc} 0.001 & 0\\ 0 & 0.091 \end{array}\right],\;\;\;R_{A}=1.6\times 10^{-6},\;R_{B}=5.97\times 10^{-5},R_{C}=1.6\times 10^{-6},\;\\ W_{1}=0.25,\;\;\;W_{2}=0.25,\;\;\;W_{3}=0.25,\;\;W_{4}=0.25. \end{array}$$

The *Q* and *R* matrices in both EKF and RKF are set as the same because the system model and measurement model are the same. Noise covariance matrices *Q* and *R* in Kalman filter implementation were treated as tuning parameters because exact values of *Q* and *R* are unknown, which is a typical remedy of Kalman filter implementation. 

## 6. Validation

For validation, we used COMSOL for the simulation of electrostatic actuator and MATLAB/Simulink for simulation of rotor dynamics. The observer and controller were implemented in MATLAB/Simulink, too. COMSOL 5.3a and MATLAB 2016 were used for simulation. COMSOL supports a tool called LiveLink so that we can easily use the connection with MATLAB. COMSOL receives the voltage value of each electrode from the controller implemented in Simulink and the rotor position value from the rotor dynamics model implemented in MATLAB. COMSOL calculates the corresponding torque value and sends it to MALAB where the rotor dynamics model is implemented.

**Figure 13:**
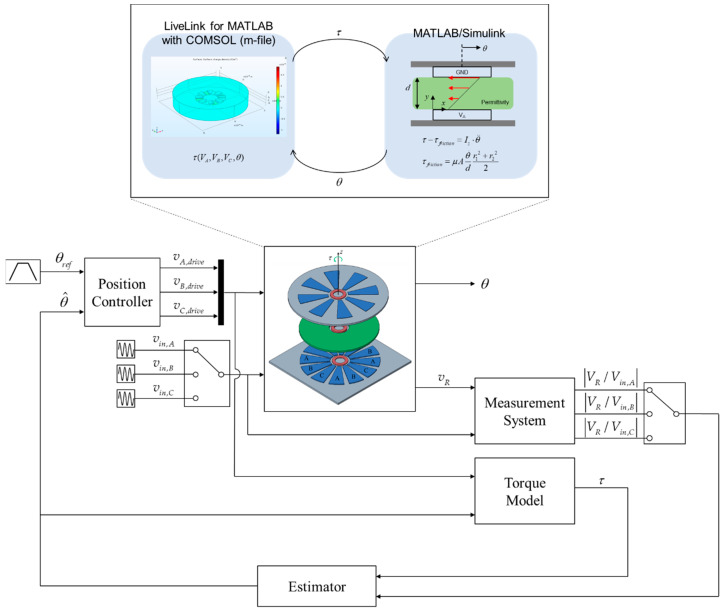
Diagram of the proposed system.

The initial values in the Simulink of position and velocity were zero. The position controller was a simple PID controller. The excitation voltage input was a sinusoidal signal with a magnitude of 0.1 V and frequency of 1 kHz. Figure 13 shows a diagram of the overall simulation. The excitation input is applied to only one electrode at one time. The electrode that should be excited for the highest accuracy is discussed in following section. 

### 6.1. Measurement Signal vs. Measurement Model

**Figure 14:**
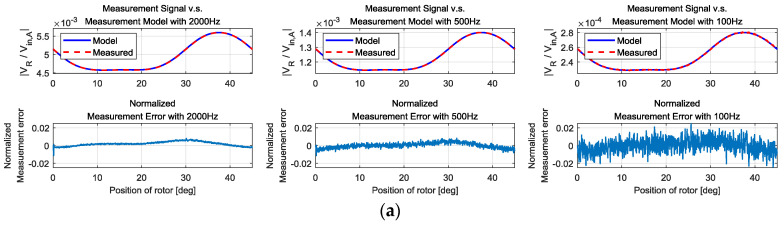

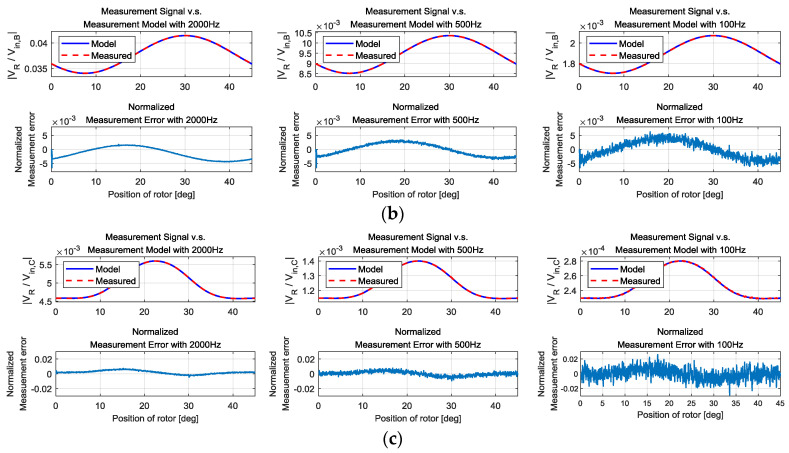
Comparison of the measurement signal and measurement model: (**a**) when *v_in,A_* is excited, (**b**) when *v_in,B_* is excited, (**c**) when *v_in,A_* is excited.

To confirm that the measurement model is valid and accurate, we performed a simulation and compared the measurement signal (the measured amplification ratio) with the evaluated value of the measurement model. Figure 14 shows the results. The measurement model shows high accuracy. The plot also shows the effect of high excitation frequency. The magnitude of the measurement changes according to the frequency. To compare the effect of the error with respect to the frequency, the normalized error is used. The normalized error is the value of error normalized by amplitude of the measurement signal. The higher the excitation frequency, the smaller the measurement error is. Another observation is that using the voltage response to *v_in,B_* would be beneficial because the signal magnitude is the biggest, which is beneficial for a large signal-to-noise ratio.

### 6.2. Rotor Position Estimation

Validation of the position estimator was also performed. The position was estimated using an EKF and UKF. There are three possible measurement signals for position estimation: the magnitude responses of (1) *v_R_* to *v_in_,_A_*, (2) *v_R_* to *v_in,B_*, and *v_R_* to *v_in,C_*, as presented in (13). We observed that the magnitude responses of *v_R_* to *v_in,B_* would be beneficial because of the large magnitude response.

**Figure 15:**
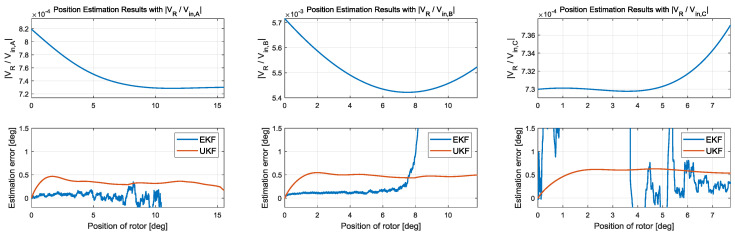
The results of position estimation comparing the EKF and UKF.

Figure 15 presents each measured signal and the corresponding estimation error. There is a time where estimation cannot be done while setting the initial estimation value because the RC circuit uses the magnitude of the high-frequency signal to measure the capacitance, so delay occurs for as much as one period of the sensing signal. Even though this delay occurs, it is for a very short time and does not significantly affect the estimation.

All three measurements have a range where the variation approaches zero. Since the EKF uses the nonlinear measurement model with a differential form, this range has a critical adverse effect on the estimation, so its estimation error diverges when the Kalman gain approaches zero due to the zero measurement sensitivity. In contrast, the UKF’s Kalman gain is less likely to approach zero because it does not use a differential value of measurement equation, so the performance evaluation of the three measurements was conducted based on the UKF.

**Figure 16:**
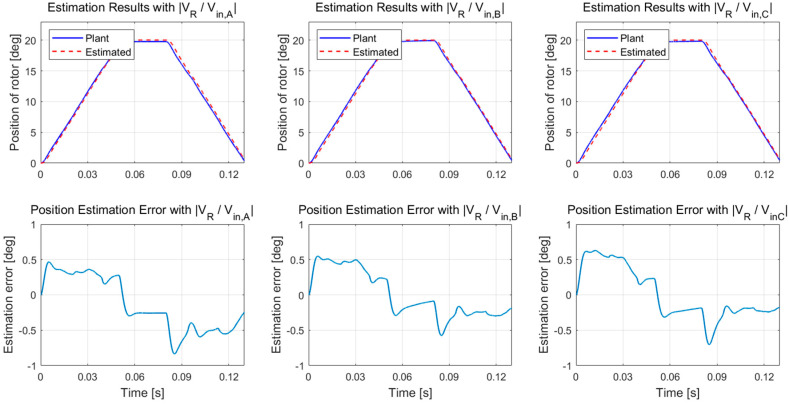
The position estimation results of each measurement of *v_R_* to *v_in,A_*, *v_in,B_*, and *v_in,C_* based on UKF.

Figure 16 shows the results of position estimation using each measurement signal. The root mean square (RMS) error of the estimator was 0.4069 degrees when the magnitude responses of *v_R_* to *v_in,A_* were used as the measurement signal. The RMS error of the case with *v_R_* to *v_in,B_* was 0.3276 degrees. The RMS error of the case with *v_R_* to *v_in,C_* was 0.3658 degrees. These results are consistent with the discussion in Section 6.1. Therefore, the best location for the excitation would be the that of *v_in,B_*.

Thus, even though the capacitance cannot be approximated as a simple flat-plate capacitor, the measurement equation based on RC circuit analysis is sufficiently accurate because of the small excitation. The EKF was not effective as an observer of the rotor position, for which the capacitance is a periodic and highly nonlinear function of rotor position. However, the UKF does not suffer from the high nonlinearity. As a result, the UKF-based estimator with the measurement signal of the magnitude responses of *v_R_* to *v_in,B_* achieved high estimation accuracy with less than 1 degree of error.

## 7. Conclusions

In this research, a position sensing system design was proposed for a MEMS electrostatic rotary actuator whose capacitance is affected by multiple electrodes and has highly nonlinear characteristics. Sensor excitation voltage and rotor driving voltage were separately applied to different electrodes for accurate capacitance measurement. Furthermore, the best location for applying the excitation signal was found to be the electrode of the sensing voltage. The UKF attenuated the instability of the observer resulting from a high nonlinearity. The proposed design could achieve high estimation accuracy in the electrostatic rotary actuator that has nonlinear capacitance. Such an approach could be applied to any other MEMS actuators that need state estimation using nonlinear capacitance. 

## References

[1] Trimmer W.S.N., Gabriel K.J. (1987). Design considerations for a practical electrostatic micro-motor. Sens. Actuators.

[2] Feng Y., Shao B., Tang X., Han Y., Wu T., Suzuki Y. (2018). Improved Capacitance Model Involving Fringing Effects for Electret-Based Rotational Energy Harvesting Devices. IEEE Trans. Electron Devices.

[3] Veroli A., Buzzin A., Frezza F., Cesare G., Hamidullah M., Giovine E., Verotti M., Belfiore N. (2018). An Approach to the Extreme Miniaturization of Rotary Comb Drives. Actuators.

[4] Pengwang E., Rabenorosoa K., Rakotondrabe M., Andreff N. (2016). Scanning Micromirror Platform Based on MEMS Technology for Medical Application. Micromachines.

[5] Park H.-C., Song C., Kang M., Jeong Y., Jeong K.-H. (2012). Forward imaging OCT endoscopic catheter based on MEMS lens scanning. Opt. Lett..

[6] Bell D.J., Lu T.J., Fleck N.A., Spearing S.M. (2005). MEMS actuators and sensors: Observations on their performance and selection for purpose. J. Micromech. Microeng..

[7] Dong J., Ferreira P.M. (2008). Simultaneous actuation and displacement sensing for electrostatic drives. J. Micromech. Microeng..

[8] Sun G., Liu T., Sen P., Shen W., Gudeman C., Kim C. (2014). Electrostatic Side-Drive Rotary Stage on Liquid-Ring Bearing. J. Microelectromech. Syst..

[9] Jacobsen S.C., Price R.H., Wood J.E., Rytting T.H., Rafaelof M. The wobble motor: An electrostatic, planetary-armature, microactuator. Proceedings of the IEEE Micro Electro Mechanical Systems, Proceedings, ‘An Investigation of Micro Structures, Sensors, Actuators, Machines and Robots’.

[10] Tang W.C., Nguyen T.H., Howe R.T. Laterally driven polysilicon resonant microstructures. Proceedings of the IEEE Micro Electro Mechanical Systems, Proceedings, ‘An Investigation of Micro Structures, Sensors, Actuators, Machines and Robots’.

[11] Legtenberg R., Groeneveld A.W., Elwenspoek M. (1996). Comb-drive actuators for large displacements. J. Micromech. Microeng..

[12] Borovic B., Lewis F.L., Liu A.Q., Kolesar E.S., Popa D. (2006). The lateral instability problem in electrostatic comb drive actuators: Modeling and feedback control. J. Micromech. Microeng..

[13] McCarthy M., Waits C.M., Beyaz M.I., Ghodssi R. A Rotary Microactuator Supported on Encapsulated Microball Bearings using an Electro-Pneumatic Thrust Balance. Proceedings of the 2009 IEEE 22nd International Conference on Micro Electro Mechanical Systems.

[14] Liang-Hsuan L., Kee Suk R., Chang L. (2002). A magnetic microstirrer and array for microfluidic mixing. J. Microelectromech. Syst..

[15] Chan M.L., Yoxall B., Park H., Kang Z., Izyumin I., Chou J., Megens M.M., Wu M.C., Boser B.E., Horsley D.A. (2012). Design and characterization of MEMS micromotor supported on low friction liquid bearing. Sens. Actuators A Phys..

[16] Yoxall B., Chan M.-L., Harake R., Pan T., Horsley D. (2012). Rotary Liquid Droplet Microbearing. IEEE/ASME J. Microelectromech. Syst..

[17] Frechette L.G., Nagle S.F., Ghodssi R., Umans S.D., Schmidt M.A., Lang J.H. An electrostatic induction micromotor supported on gas-lubricated bearings. Proceedings of the 14th IEEE International Conference on Micro Electro Mechanical Systems (Cat. No.01CH37090).

[18] Chee Wei W., Xin Z., Jacobson S.A., Epstein A.H. (2004). A self-acting gas thrust bearing for high-speed microrotors. J. Microelectromech. Syst..

[19] Lee J., Huang X., Chu P.B. (2009). Nanoprecision MEMS Capacitive Sensor for Linear and Rotational Positioning. J. Microelectromech. Syst..

[20] Moore S.I., Moheimani S.O.R. (2014). Simultaneous Actuation and Sensing for Electrostatic Drives in MEMS using Frequency Modulated Capacitive Sensing. Ifac Proc. Vol..

[21] Chen Y., Aktakka E.E., Woo J.-K., Najafi K., Oldham K.R. (2018). On-chip capacitive sensing and tilting motion estimation of a micro-stage for in situ MEMS gyroscope calibration. Mechatronics.

[22] Chen Y., Li H., Qiu Z., Wang T.D., Oldham K.R. (2020). Improved Extended Kalman Filter Estimation Using Threshold Signal Detection With an MEMS Electrostatic Microscanner. Ieee Trans. Ind. Electron..

[23] Kambali P.N., Pandey A.K. (2016). Capacitance and Force Computation Due to Direct and Fringing Effects in MEMS/NEMS Arrays. IEEE Sens. J..

[24] Leus V., Elata D. (2004). Fringing Field Effect in Electrostatic Actuators.

[25] Hammer H. (2010). Analytical Model for Comb-Capacitance Fringe Fields. J. Microelectromech. Syst..

[26] Moore S.I., Moheimani S.O.R. A switched actuation and sensing method for a MEMS electrostatic drive. Proceedings of the 2016 American Control Conference (ACC).

[27] He J., Xie J., He X., Du L., Zhou W., Wang L. (2014). Calculating capacitance and analyzing nonlinearity of micro-accelerometers by Schwarz–Christoffel mapping. Microsyst. Technol..

[28] Sun T., Green N.G., Morgan H. (2008). Electric field analysis using Schwarz-Christoffel mapping. J. Phys. Conf. Ser..

[29] Bruschi P., Nannini A., Pieri F., Raffa G., Vigna B., Zerbini S. (2004). Electrostatic analysis of a comb-finger actuator with Schwarz–Christoffel conformal mapping. Sens. Actuators A Phys..

[30] Palmer H.B. (1937). The capacitance of a parallel-plate capacitor by the Schwartz-Christoffel transformation. Electr. Eng..

[31] Hoshiya M., Saito E. (1984). Structural Identification by Extended Kalman Filter. J. Eng. Mech..

[32] Wan E.A., Merwe R.V.D. The unscented Kalman filter for nonlinear estimation. Proceedings of Proceedings of the IEEE 2000 Adaptive Systems for Signal Processing, Communications, and Control Symposium (Cat. No.00EX373).

[33] Julier S.J., Uhlmann J.K. (2004). Unscented filtering and nonlinear estimation. Proc. IEEE.

